# Gallic Acid Alleviates Glucolipotoxicity-Induced Nephropathy by miR-709-NFE2L2 Pathway in db/db Mice on a High-Fat Diet

**DOI:** 10.1021/acs.jafc.4c05898

**Published:** 2024-10-04

**Authors:** Ang-Tse Lee, Mon-Yuan Yang, I-Ning Tsai, Yun-Ching Chang, Tung-Wei Hung, Chau-Jong Wang

**Affiliations:** † Institute of Medicine, Chung Shan Medical University, Taichung 402, Taiwan; ‡ Department of Health Industry Technology Management, Chung Shan Medical University, Taichung 402, Taiwan; § Department of Medical Research, Chung Shan Medical University Hospital, Taichung 402, Taiwan; ∥ Division of Nephrology, Department of Medicine, Chung Shan Medical University Hospital, Taichung 40201, Taiwan; ⊥ School of Medicine, Chung Shan Medical University, Taichung 402, Taiwan

**Keywords:** oxidative stress, gallic acid, diabetic nephropathy, NFE2L2 pathway

## Abstract

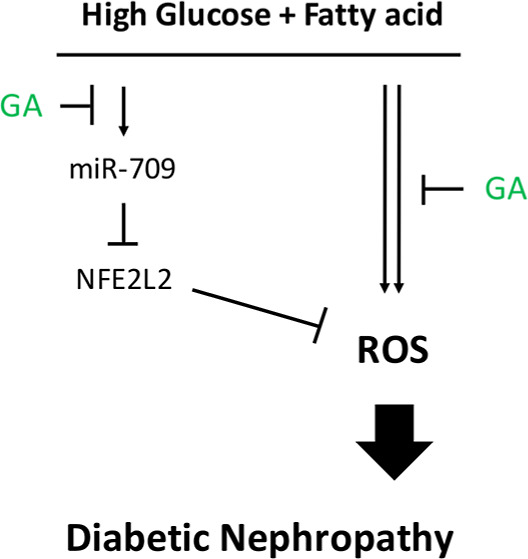

Background: Type 2 diabetes mellitus (T2DM) has become a major global issue, with diabetic nephropathy (DN) ranking as one of its most serious complications. The involvement of microRNAs (miRNAs) in the progression of T2DM and DN is an area of active research, yet the molecular mechanisms remain only partially elucidated. Gallic acid (GA), a naturally occurring polyphenolic compound found in various plants such as bearberry leaves, pomegranate root bark, tea leaves, and oak bark, has demonstrated antioxidant properties that may offer therapeutic benefits in DN. Methods and Results: The study aimed to investigate the therapeutic potential of GA in mitigating kidney fibrosis, oxidative stress and inflammation, within a glucolipotoxicity-induced diabetic model using db/db mice. The mice were subjected to a high-fat diet to induce glucolipotoxicity, a condition that mimics the metabolic stress experienced in T2DM. Through microarray data analysis, we identified a significant upregulation of renal miR-709a-5p in the diabetic mice, linking this miRNA to the pathological processes underlying DN. GA treatment was shown to boost the activity of including catalase, essential antioxidant enzymes, glutathione peroxidase and superoxide dismutase, while also reducing lipid accumulation in the kidneys, indicating a protective effect against HFD-induced steatosis. In vitro experiments further revealed that silencing miR-709a-5p in MES-13 renal cells led to a reduction in oxidative stress markers, notably lowering lipid peroxidation markers, and significantly boosting the activity of antioxidant defenses. Additionally, NFE2L2, a crucial transcription factor involved in the antioxidant response, was identified as a direct target of miR-709a-5p. The downregulation of miR-709a-5p by GA suggests that this polyphenol mitigates glucolipotoxicity-induced lipogenesis and oxidative stress, potentially offering a novel therapeutic avenue for managing diabetic fatty liver disease and DN. Conclusion: Our findings indicate that GA exerts a protective effect in DN by downregulating miR-709a-5p, thereby alleviating oxidative stress through the suppression of NFE2L2. The results highlight the potential of GA and NFE2L2-activating agents as promising therapeutic strategies in the treatment of DN.

## Introduction

Diabetic nephropathy (DN) is a complication of diabetes that results from microvascular problems such as microangiopathy. This progressive condition causes a decline in kidney function and is common among patients with diabetes mellitus (DM). DN is a major contributor to end-stage renal disease worldwide because of its negative effect on the kidneys.^34^ Glucolipotoxicity is another major complication of diabetes that involves kidney damage through elevated glucose and lipid levels.

The discovery that small noncoding RNAs control gene expression has transformed biology. Advancements in technology have enabled identification of various ncRNAs, including small interfering RNAs and microRNAs (miRNAs). miRNAs are main regulators of gene expression, primarily at the posttranscriptional level. Studies have explored the role miRNAs play in the nucleus, particularly that with respect to regulating gene expression at the transcriptional level, although the mechanisms of this regulation remain to be elucidated. Such studies have focused on identifying the nuclear functions of miRNAs and how they recognize and silence genes at the promoter level.^4^ miRNAs are small ncRNA molecules that regulate gene expression and thus affect cellular processes such as cell cycle progression and apoptosis. Their role is not limited to actions in cytoplasm; it includes actions in the nuclear.^28^ According to the literature, miRNAs are involved in the pathogenesis of many human disease^8,44^ because they play a role in the control of biological functions associated with disease progression, such as apoptosis, proliferation, development and differentiation.^16,30^ Many studies have analyzed miRNAs and their correlations with various diseases, such as cancer in different organs.^37,42^ According to recent studies, miRNAs play a key role in renal development, renal function maintenance, and kidney disease progression.^10,26,37^ Multiple pathways have been identified as contributors to the pathogenesis of renal disease, such as the TGF-β, MAPK, and Wnt signaling pathways.^3,5,31^ According to multiple studies, miRNAs play a key role in kidney function in conditions such as renal cell carcinoma,^25^ DN,^18^ polycystic kidney disease,^9^ IgA nephropathy,^43^ acute kidney injury (AKI) related to ischemia–reperfusion injury,^2^ and lupus nephriti^22^ by influencing the processes of apoptosis, cell proliferation, and differentiation. miRNAs are detectable in biological fluids, and they can be used to elucidate disease pathogenesis and diagnosis, which can offer valuable insights into kidney pathologies that can enable development of novel therapeutic approaches. In renal proximal tubular cells, the expression of miR-709 mediates acute tubular injury by affecting mitochondrial function.^12^

Gallic acid (GA) is commonly present in a variety of plants, including bearberry leaves, pomegranate root bark, gallnuts, witch hazel, sumac, tea leaves, oak bark, and others, either in its free form or incorporated within tannin molecules. Regarded as a natural antioxidant, GA is essentially a secondary polyphenolic metabolite. It plays a key role in traditional Ayurvedic remedies, and it is particularly common in antioxidant tea formulations.^17^ Studies on the antihyperlipidemic effects of GA in mice fed a high-fat diet (HFD) suggest it leads to a decrease in triglycerides and low-density lipoprotein cholesterol levels, while promoting an increase in high-density lipoprotein cholesterol. In streptozotocin-induced diabetic rats, GA was reported to have a protective effect across several biochemical and histopathological parameters.^11^ We analyzed the effects of GA on kidney fibrosis, inflammation, and oxidative stress in a glucolipotoxicity-induced diabetic db/db mouse model. Glucolipotoxicity was prompted by feeding the mice an HFD. Subsequently, microarray data analysis was conducted to identify the affected miRNAs and their target signals and determine how miRNAs regulate the effects and mechanisms of DN complications in diabetes. We explored whether GA affects the expression of miRNAs and their target signals. We hypothesize that GA improves DN by downregulating miR-709a-5p, leading to enhanced antioxidant defenses via NFE2L2 suppression. Additionally, we systematically analyzed the illustration and function of miRNA genes in mice with Type 2 diabetes mellitus (T2DM) to establish the role of miRNAs in diabetes-induced nephropathy and to identify the mechanisms through which polyphenolic substances like GA regulate miRNAs.

## Materials and Methods

### Animals Maintenance and Treatment

The original population of BKS.Cg-Dock7m+/+ Leprdb/JNarl (db/db) mice, weighing approximately 20 g at 4 to 6 weeks of age, was acquired from The Jackson Laboratory and was maintained at the National Laboratory Animal Center in Taipei, Taiwan. The mice were maintained at 22 ± 2 °C in an environment that was well-organized and had a 12 h light/dark cycle. The Chung Shan Medical University Institutional Animal Care and Use Committee approved all experimental protocols (IACUC, CSMU, approval no. 2025). The db/db mice, a model for type 2 diabetes, were used in this study, with db/m heterozygous mice serving as age-matched controls. The mice were separated to four groups: (1) db/m control group (*n* = 3), fed with a Standard Laboratory Rodent Diet 5010 (24.6% protein, 6.1% ash, 5.0% fat, and 4.2% crude fiber) for 12 weeks; (2) db group (*n* = 3; db/db mice fed the same standard diet for 12 weeks); (3) HFD (*n* = 3; db/db mice fed a HFD [0.5% cholesterol and 15% lard oil] for 12 weeks); and (4) GA group (*n* = 3; db/db mice fed a HFD along with intragastric administration of GA at 100 mg/kg for 12 weeks). Through heart puncture, we obtained blood samples for biochemical analysis, and kidneys were removed to determine weight and evaluate damage using pathological inspection.

### Plasma Biomarkers for Kidney Function and Biochemical Analysis

Using commercially available kits from Randox Laboratories, enzymatic colorimetric techniques were used to measure the plasma biochemical levels of blood urea nitrogen (BUN; UR107, Randox Laboratories, Antrim, UK), creatinine (CR510, Randox Laboratories), and uric acid (UA230, Randox Laboratories). Fortress Diagnostics (UK) kits were used to measure glycosylated hemoglobin (HbA1c) levels, whereas Mercodia AB (Uppsala, Sweden) kits were used to measure insulin levels.

### Extent of Antioxidant Enzymes and Lipid Peroxidation

By measuring the amount of malondialdehyde that reacted with thiobarbituric acid to form thiobarbituric acid reactive substances (TBARS), lipid peroxidation was evaluated. At 532 nm, the supernatant’s absorbance was measured. Protein values for TBARS are expressed as mmol/mg. The assay mixture’s glutathione (GSH) content was quantified both prior to and following enzymatic activity (g/mg protein). The method described by Lawrence and Burk was used to spectrophotometrically determine the GSH peroxidase (GSH Px) activity (nmol NADPH/min/mg protein). Utilizing phosphate-buffered saline (containing 1.1 mM MgCl2·6H2O, 5.0 mM glutathione disulfide, and 0.1 mM NADPH) at 340 nm, GSH reductase (GSH Rd) activity (nmol NADPH/min/mg protein) was measured strictly. The expected level of superoxide dismutase (SOD) activity (U/mg protein) was determined by inhibiting pyrogallol autoxidation at a concentration of 0.2 mM (*U* = 50% inhibition of activity). Three mM H_2_O_2_ was used to assess the catalase activity (U/mg protein) at 240 nm.

### Western Blotting Analysis

After the cells were treated with the reagents for 24 h, they were lysed using RIPA lysis buffer. After centrifuging the lysates, the supernatant was gathered and put aside for examination. Using sodium dodecyl sulfate–polyacrylamide gel electrophoresis, 50 μg of protein samples were separated and then placed onto nitrocellulose membranes (Millipore, Bedford, MA, USA). The appropriate primary antibody was then incubated overnight at 4 °C on the blocked membranes, which had first been blocked with a solution of 5% nonfat milk powder in Tris-buffered saline (TBS) containing 0.1% Tween 20. Following incubation, the membranes were repeatedly washed with TBS containing 0.1% Tween 20, and then an incubation was conducted using a secondary antibody conjugated with horseradish peroxidase (GE Healthcare, Little Chalfont, Buckinghamshire, UK). The Fujifilm LAS-4000 system (Tokyo, Japan) was utilized to identify protein bands on ECL hyperfilm and visualize them using enhanced chemiluminescence (ECL). Fujifilm-Multi Gauge V2.2 software was used to perform densitometry analysis for protein quantification (Tokyo, Japan).

### Histological Examination of the Tissues

Kidney specimens were collected and fixed in 10% neutral buffered formalin for Masson’s trichrome staining, hematoxylin and eosin (H&E) staining, immunohistochemical analysis utilizing the specific marker 8-hydroxy-2′-deoxyguanosine (8-OHdG) for oxidative injury and staining with Periodic acid-Schiff (PAS). Hematoxylin was first applied to the slides for 30 s in order to stain them with H&E. They were soaked in water, dyed for two to 5 min with eosin, and then dehydrated using a graduated alcohol series. Tissues were fixed in Bouin’s solution before being rinsed in water for 10 min to remove the yellow tint in Masson’s trichrome staining. Weigert’s iron hematoxylin was applied to them for approximately 10 min, followed by a 10 min warm water washing. After that, the sections were differentiated in a phosphomolybdic-phosphotungstic acid solution for 5 min and stained for 7 min with Biebrich scarlet-acid fuchsin. The pieces were separated in 1% acetic acid for 3 min and then briefly washed in distilled water following their 5 min exposure to aniline blue. To get rid of extra Biebrich scarlet-acid fuchsin staining, the sections were successively dehydrated with 50%, 75%, 95%, and 100% alcohol. After that, they were cleaned with xylene. The glomeruli’s histological alterations were then seen under a light microscope.

### Cell Treatment

To cause renal damage, a combination of 100 μM palmitic acid (PA) and 25 mM high glucose (HG) was applied to murine glomerular mesangial (MES-13) cells. Higher glucose levels from HG cause more ROS to be produced; PA, a saturated fatty acid, increases ROS generation in mitochondria and triggers inflammatory pathways, both of which contribute to oxidative stress. GeneDireX (Las Vegas, NV, USA) provided the negative control, miRNA mimics, and miRNA inhibitors. T-Pro nonliposomal transfection reagent II (T-Pro NTR II, T-Pro Biotechnology, Taipei, Taiwan) was used for the transfection process.

### Real-Time Polymerase Chain Reaction and miRNA Isolation

Renal tissues were used to extract total RNA, which was then converted into cDNA. Next, RT-PCR, or stem-loop reverse transcription polymerase chain reaction, was carried out. The LightCycler 480 SYBR Green I Master mix was used in real-time PCR utilizing a Light Cycler 480 real-time PCR equipment (Roche Applied Science). The levels of miRNA expression were adjusted in relation to RNU6B (6B), an internal reference. The 2^–Δ*Ct*^ technique was used to analyze the data after each reaction was done three times.

The reverse transcription primers (5′–3′) utilized for miR-709 and 6B, respectively, were GTTGGCTCTGGTGCAGGGTCCGAGGTATTCGCACCAGAGCCAACTCCTCC and GTGCAGGGTCCGAGGTATTCGCACCAGAGCCAACAAAAATAT.

GGAGGCAGAGGCAGGA CGATTGGCAGTGTCTTAGCT, miR-709 forward; TTCCTCCGCAAGGATGACACGC; and GTGCAGGGTCCGAGGT, universal reverse primer, were the primers (5′–3′) utilized for real-time PCR.

### Luciferase Reporter Assay

Using miRNA 3′-UTR target constructs from GeneCopoeia’s miTargetTM system—which is intended for miRNA identification and functional validation of predicted targets—luciferase activity was measured. A secreted Gaussia luciferase (GLuc) reporter gene was combined with 3′-UTR sequences that were obtained from a gene sequence database. The 3′-UTR target sequence and the GLuc reporter were present in the resulting chimeric mRNA. The relationship between mRNA and miRNA was next evaluated using a live-cell assay, which required just 10 μL of cell culture media to detect GLuc. An internal control, secreted alkaline phosphatase (SEAP), was cloned and used as the miRNA 3′-UTR target reporter GLuc. To confirm that the 3′-UTR sequence of NFE2L2 (gene accession no. NM_001145413.2) interacts with miR-709, this dual-reporter system (GLuc and SEAP) was used.

### Statistical Analysis

The mean ± standard deviation (SD) was used to represent the data. Group differences were assessed using analysis of variance (ANOVA), with *p* < 0.05 designated as the statistical significance level. To find significant differences across groups, Duncan’s multiple range test (Sigma-Plot 12.0, Jandel Scientific, San Rafael, CA, USA) was used.

## Results

### GA Improves Renal Function and Ameliorates Hyperuricemia, Hyperinsulinemia, and Glomerular Damage in Diabetic Mice

**Figure 1:**
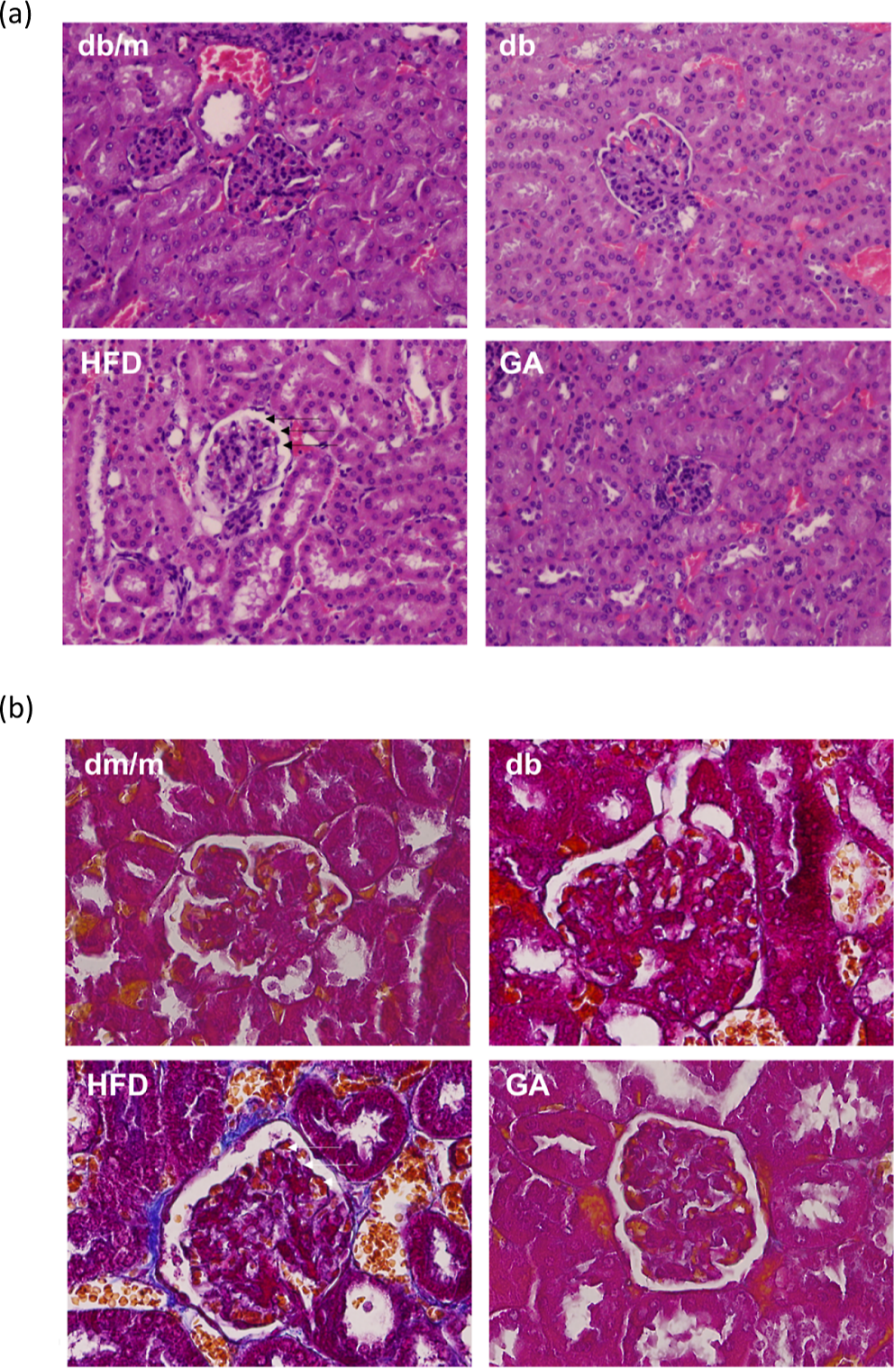
HFD induced significant pathological changes in the glomeruli of diabetic mice and GA improved it. The 6 week-old male db/db mice fed with HFD and 100 mg/kg GA for 12 weeks. (a) H&E (200×) and (b) Masson’s trichrome staining (400×) of renal frozen sections. The glomerulus is indicated with an arrow. db/m, age-matched heterozygous mice fed with standard diet; db, db/db mice fed with standard diet group; HFD, db/db mice fed with HFD; GA, db/db mice fed with HFD and GA.

**Figure 2:**
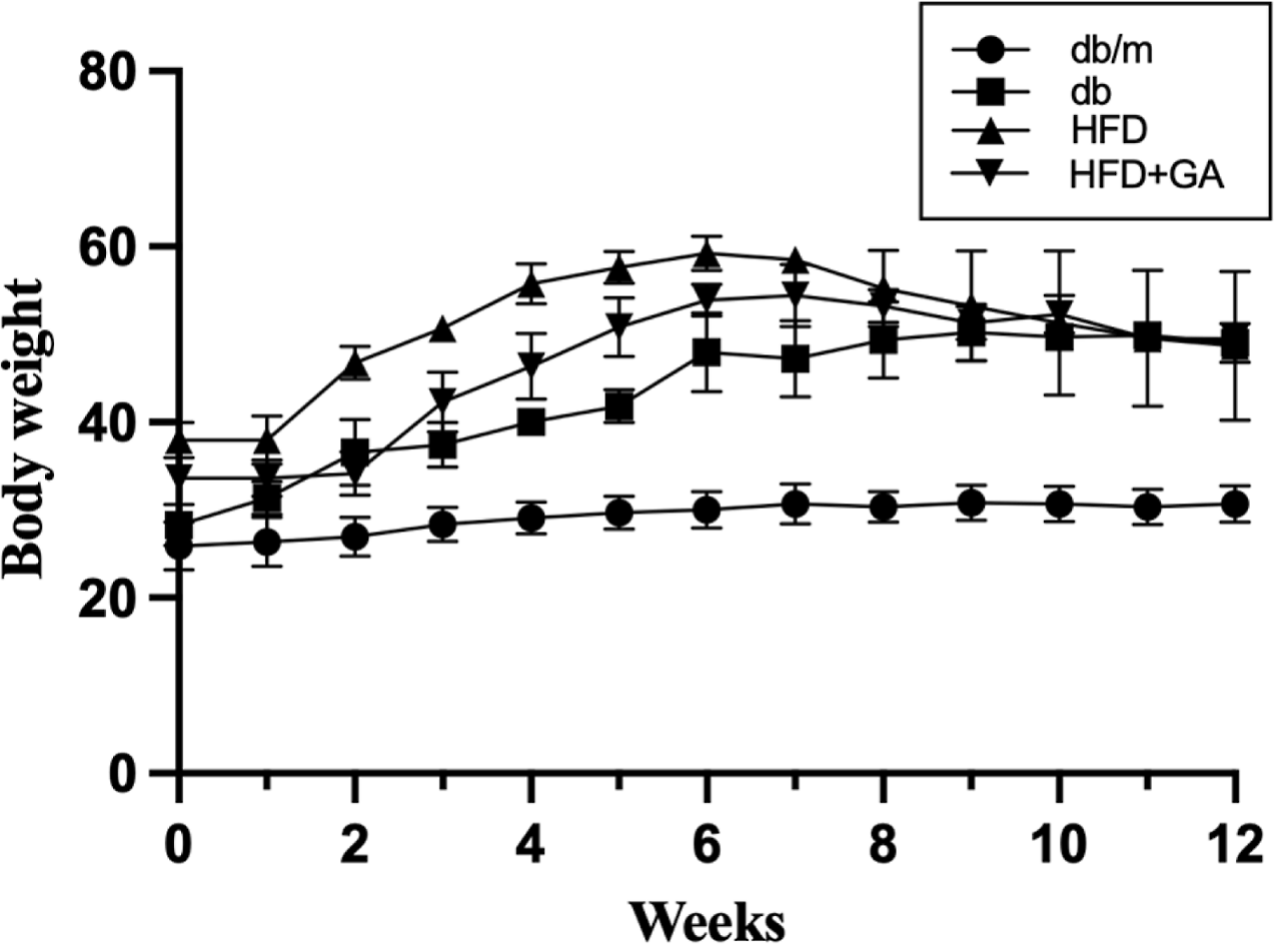
Body weight changes of mice during feeding experimental period. db/m, age-matched heterozygous mice fed with standard diet; db, db/db mice fed with standard diet; HFD, db/db mice fed with HFD; GA, db/db mice fed with HFD and GA. Each value is expressed as the mean ± SD (*n* = 3/group). Results were statistically analyzed with ANOVA. Unit of body weight: gram. ^a^, *p* < 0.05 compared with the db/m group. ^b^, *p* < 0.05 compared with the db group. ^c^, *p* < 0.05 compared with the HFD group.

To determine the effects of an HFD and GA on the kidneys of mice, we analyzed various plasma biochemical parameters and histopathological changes. In this study, we utilized four groups to test our hypothesis. The groups included: db/m mice as the negative control, db/db mice as the control group, db/db mice with a HFD representing the T2DM group, and db/db mice with HFD treated with GA as the treatment group. We discovered that compared to the db group of mice, the HFD group had upper plasma levels of BUN, creatinine, and insulin (Table 1). The results showed that an HFD was associated with weakened renal function and hyperinsulinemia. Compared with the HFD group, the GA-treated group shown a greater upgrading in renal parameters and uric acid levels. In addition, consuming an HFD resulted in basement membrane thickening in the glomerulus and a marked deposition of collagen, as observed using H&E staining and Masson’s trichrome staining, individually (Figure 1). These results indicated that the HFD caused structural changes in the glomeruli and induced deposition of collagen fibers accompanied by a decline in renal function. In diabetic mice in which GA was administered for 12 weeks, the degree of basement membrane thickening and collagen deposition was attenuated, indicating GA has renoprotective effects against HFD-induced kidney injury. In summary, GA ameliorated nephropathy by restoring renal function and protecting against hyperinsulinemia, basement membrane thickening, and glomerular fibrosis in HFD-fed mice. Through the 12 weeks of the feeding experiment, the body weight changes of the four groups of mice were recorded (Figure 2). We indicated that the body weight of the db/db mice on a standard diet was upper than that of the heterozygous mice on the same diet. In the db/db mice, during the feeding experiment, HFD feeding further increased their body weight, whereas GA treatment reduced it.

**Table 1:** Plasma Renal Parameters and Kidney Weight in Mice^a^

	db/m	db	HFD	GA
BUN (mg/dL)	12.83 ± 1.91	13.98 ± 2.54	28.03 ± 4.68^b^^,^^c^	14.97 ± 4.42^c^^,^^d^
CRE (mg/dL)	0.42 ± 0.04	0.48 ± 0.04	0.60 ± 0.13^b^^,^^c^	0.43 ± 0.05^d^
UA (mg/dL)	4.38 ± 0.33	7.35 ± 1.01^b^	7.85 ± 1.43	6.35 ± 0.87^b^^,^^c^^,^^d^
HbA1c (%)	4.47 ± 0.71	8.23 ± 1.40^b^	8.30 ± 0.88^b^	8.10 ± 1.33^b^
Insulin (pg/L)	1.03 ± 0.09	5.24 ± 0.37^b^	7.26 ± 1.83^b^^,^^c^	5.20 ± 0.51^b^^,^^c^
Kidney weight (g)	0.46 ± 0.02	0.41 ± 0.01	0.44 ± 0.01	0.43 ± 0.02

a db/m, age-matched heterozygous mice fed with standard diet; db, db/db mice fed with standard diet; HFD, db/db mice fed with HFD; GA, db/db mice fed with HFD and GA. BUN, blood urea nitrogen. CRE, creatinine. UA, uric acid. HbA1c, glycosylated hemoglobin. Each value is expressed as the mean ± SD (*n* = 3/group). Results were statistically analyzed with ANOVA.

b *p* < 0.05 compared with the db/m control group.

c *p* < 0.05 compared with the db group.

d *p* < 0.05 compared with the HFD group.

### HFD Induces Renal Lipid Peroxidation in Diabetic Mice

**Figure 3:**
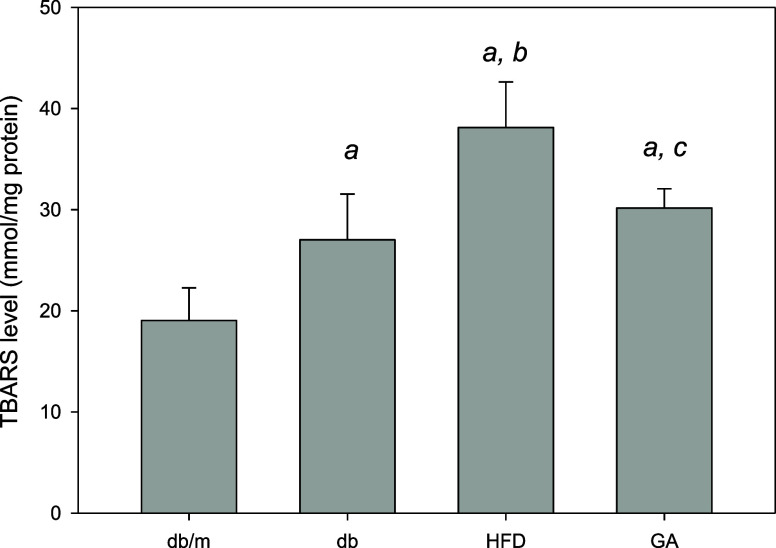
HFD induced renal lipid peroxidation and GA ameliorated it in diabetic mice. The 6 week-old male db/db mice fed with HFD and 100 mg/kg GA for 12 weeks. TBARS levels of the kidney were analyzed. db/m, age-matched heterozygous mice fed with standard diet; db, db/db mice fed with standard diet; HFD, db/db mice fed with HFD; GA, db/db mice fed with HFD and GA. ^a^, *p* < 0.05 compared with the db/m control group. ^b^, *p* < 0.05 compared with the db group. ^c^, *p* < 0.05 compared with the HFD group.

In Figure 3, the TBARS levels, which are symbols of lipid peroxidation, were significantly elevated in the kidneys of db/db mice with a HFD compared to the db/m control group, indicating that the HFD induced substantial oxidative stress. However, when these HFD-fed mice were treated with 100 mg/kg of GA for 12 weeks, there was a significant reduction in TBARS levels, suggesting that GA effectively ameliorated lipid peroxidation in the kidneys.

### HFD Induces Renal Oxidative Injury in db/db Mice

**Figure 4:**
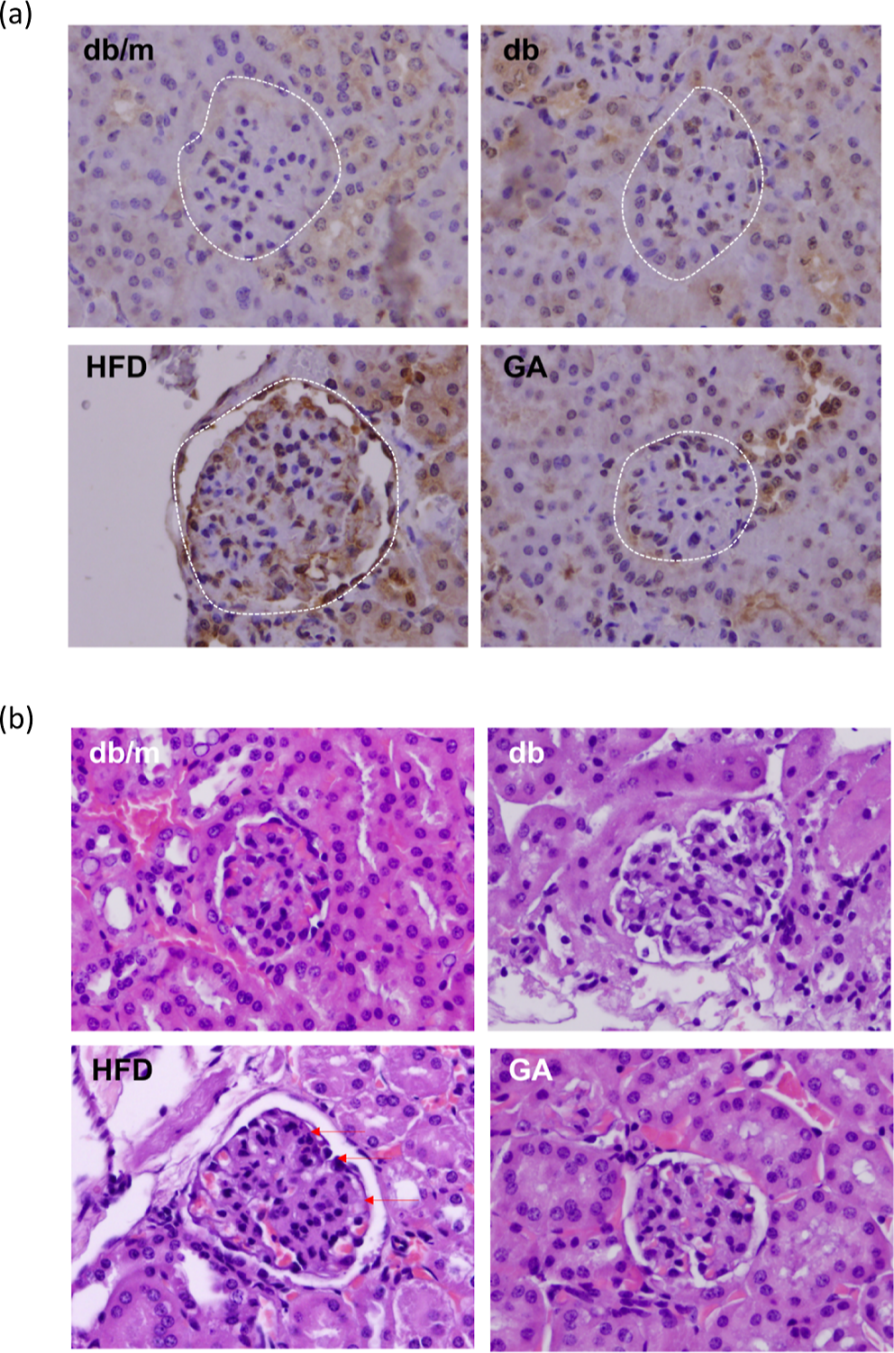
The 6 week-old male db/db mice fed with HFD and 100 mg/kg GA for 12 weeks. (a) HFD induced renal oxidative injury and GA improved it in the glomeruli in diabetic mice. Renal tissue samples were obtained for determination of specific marker 8-OHdG antibody (for oxidative injury) using immunohistochemical analysis (400×). The glomerulus is indicated with a circle of white line. (b) Histological staining with PAS in the glomeruli (400×). HFD induced basement membrane thickening (arrow) and glycogen accumulation in the glomerulus and GA improved it.

We further explored the protective effects of GA on renal oxidative injury (Figure 4). Immunohistochemical analysis using the 8-OHdG antibody revealed that HFD-fed db/db mice exhibited marked oxidative damage in the glomeruli, which is a critical structure in the kidney. The damage was characterized by increased staining, indicating higher levels of oxidative stress markers. However, in the GA-treated group, there was a noticeable reduction in oxidative damage within the glomeruli, demonstrating the ability of GA to mitigate oxidative stress. Additionally, histological analysis using PAS staining showed that HFD induced basement membrane thickening and glycogen accumulation in the glomeruli, both of which are indicative of kidney damage. GA treatment significantly improved these pathological changes, reducing both membrane thickening and glycogen accumulation.

### HFD Induces Renal Antioxidant Enzyme Activities in Diabetic Mice

**Figure 5:**
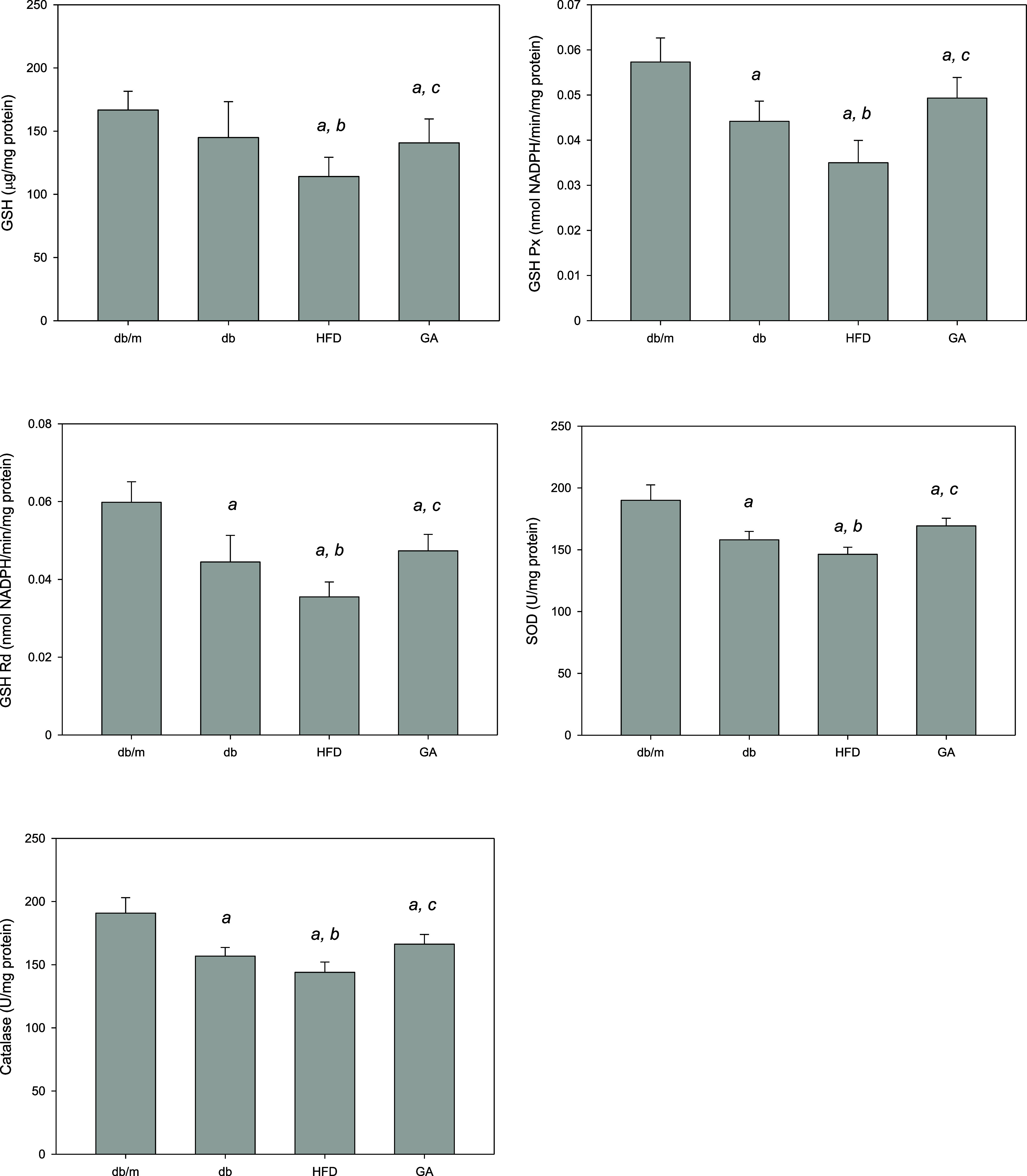
HFD reduced and GA enhanced the activities of renal antioxidant enzymes in diabetic mice. The 6 week-old male db/db mice fed with HFD and 100 mg/kg GA for 12 weeks. Activity levels of GSH, GSH Px, GSH Rd, catalase, and SOD in the kidney were analyzed. db/m, age-matched heterozygous mice fed with standard diet; db, db/db mice fed with standard diet; HFD, db/db mice fed with HFD; GA, db/db mice fed with HFD and GA. ^a^, *p* < 0.05 compared with the db/m control group. ^b^, *p* < 0.05 compared with the db group. ^c^, *p* < 0.05 compared with the HFD group.

Then we focused on the actions of renal antioxidant enzymes, including GSH, GSH Px, GSH Rd, catalase, and SOD, in the different groups of mice (Figure 5). The HFD led to a noteworthy reduction in the activities of these enzymes in the kidneys of diabetic mice, highlighting the oxidative burden imposed by the diet. However, treatment with GA resulted in a substantial enhancement of these antioxidant enzyme activities, bringing them closer to the levels detected in the db/m control group. This advises that GA not only reduces oxidative damage but also bolsters the kidney’s antioxidant defenses. Overall, these findings indicate that GA has a defensive role against the oxidative stress and kidney damage induced by a HFD in diabetic mice, making it a potential therapeutic agent for mitigating DN.

### HFD Induces miRNA Expression in db/db Mice

**Figure 6:**
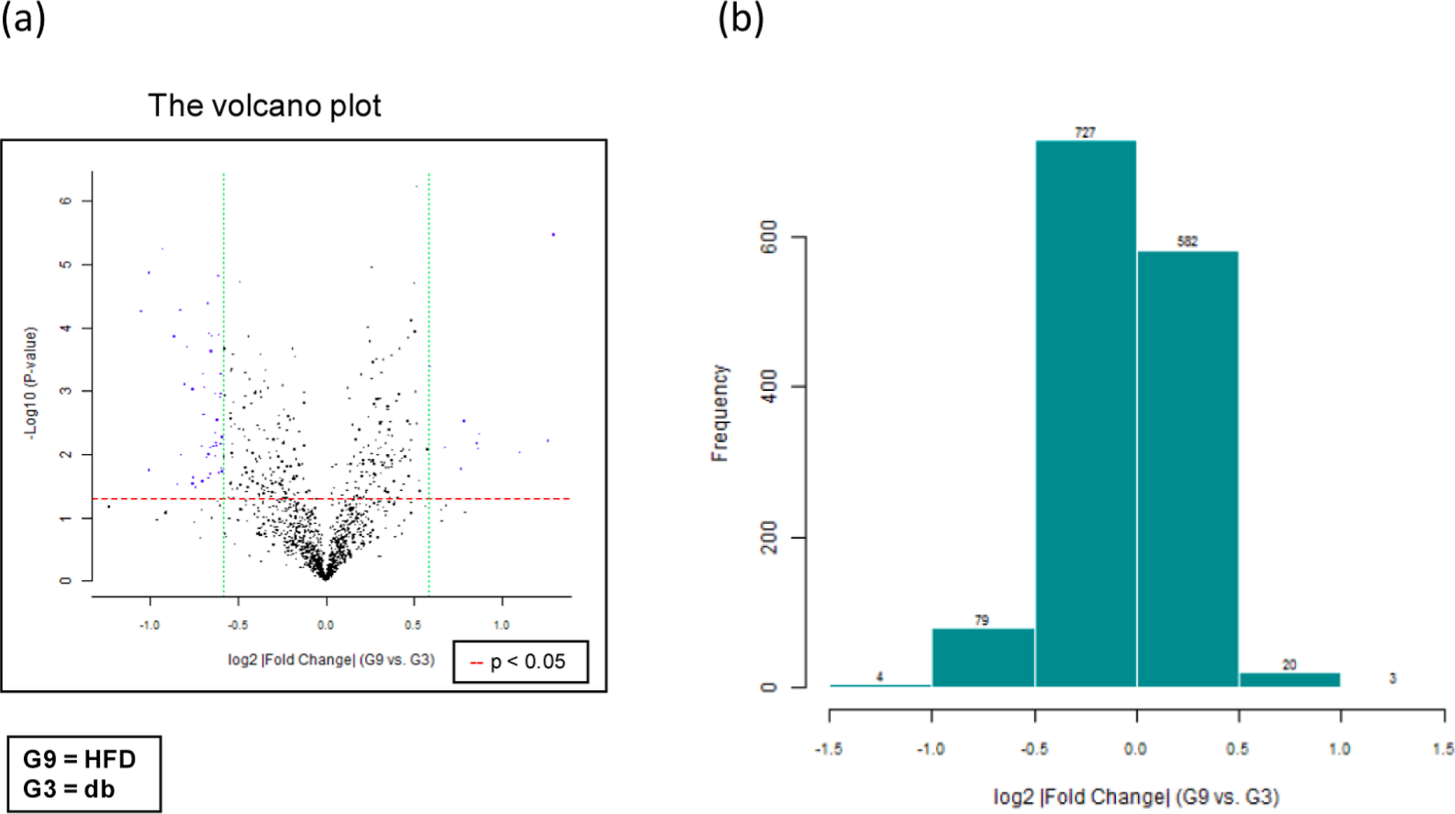
miRNA microarray validation with qRT-PCR analysis in renal tissue samples from grouped HFD-fed db/db mice and standard diet-fed db/db mice. The 6 week-old male db/db mice were fed with HFD for 12 weeks. After euthanization, renal tissue samples were obtained for the determination of miRNA expression. miRNAs were selected from the grouped miRNA microarray data set and examined through qRT-PCR. The miRNA was sorted on the basis of log 2 fold changes in miRNA expression and are represented as horizontal bars (red line in figure). (a) The volcano plot of renal miRNA expression in HFD (G9) versus db (G3). Standard selection criteria to identify differentially expressed genes were established at log 2 |fold change| ≥ 0.585 and a *p*-value of <0.05 (blue dots in figure). (b) Histogram of log 2 |fold change| (HFD versus db). Error bars represent the SD of the mean ± SD db, db/db mice fed with standard diet; HFD, db/db mice fed with HFD.

**Figure 7:**
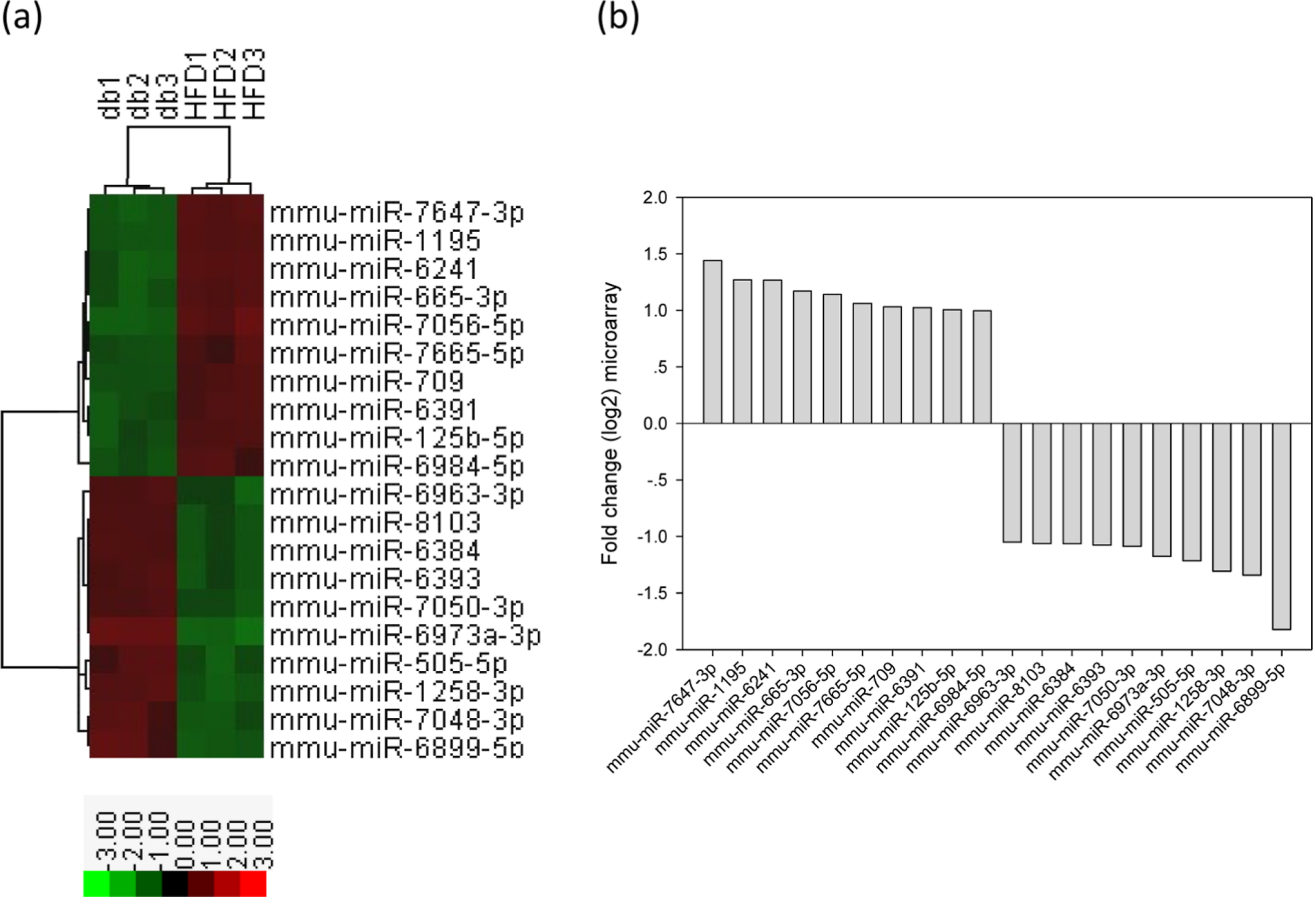
Top 10 upregulated and downregulated miRNA expression in the renal tissue from HFD-fed db/db mice. (a) A subset of differential genes was selected for clustering analysis. (b) Representation of top 10 upregulated and downregulated genes in red and green colors. miRNAs were detected both by microarray analysis between HFD-fed db/db mice and standard diet-fed db/db mice. The corresponding log 2 fold changes in miRNA abundance for each miRNA expression level as determined by microarray analysis are shown.

Both miRNA microarray authentication (Mouse & Rat miRNA OneArray; Phalanx Biotech Group, Hsinchu, Taiwan) and quantitative reverse transcription polymerase chain reaction (qRT-PCR) analysis were used to compare the expression of miRNAs in db/db mice fed with HFD and a standard diet. Kidney tissue samples were gained to quantify the expression of miRNAs. To compare the miRNA expression patterns of the HFD and standard diet groups, miRNAs were designated from a grouped miRNA microarray data set and analyzed using qRT-PCR. As presented in Figure 6a, these miRNAs were ranked based on log 2-fold changes in expression and displayed as horizontal bars (red line). To identify differentially expressed genes, the selection criteria were set at a log 2 |fold change| ≥ 0.585 and a *p*-value <0.05 (represented by blue dots) when comparing the HFD (G9) and db (G3) groups. Figure 6b displays histograms illustrating the log 2 |fold change| (HFD vs db) along with the corresponding gene counts. Figure 7 shows a heatmap of the top 10 up- and down-regulated genes related to kidney function in the HFD-fed mice.

### Identify miRNA Target Genes Using Web-Based Bioinformatics Analysis

**Figure 8:**
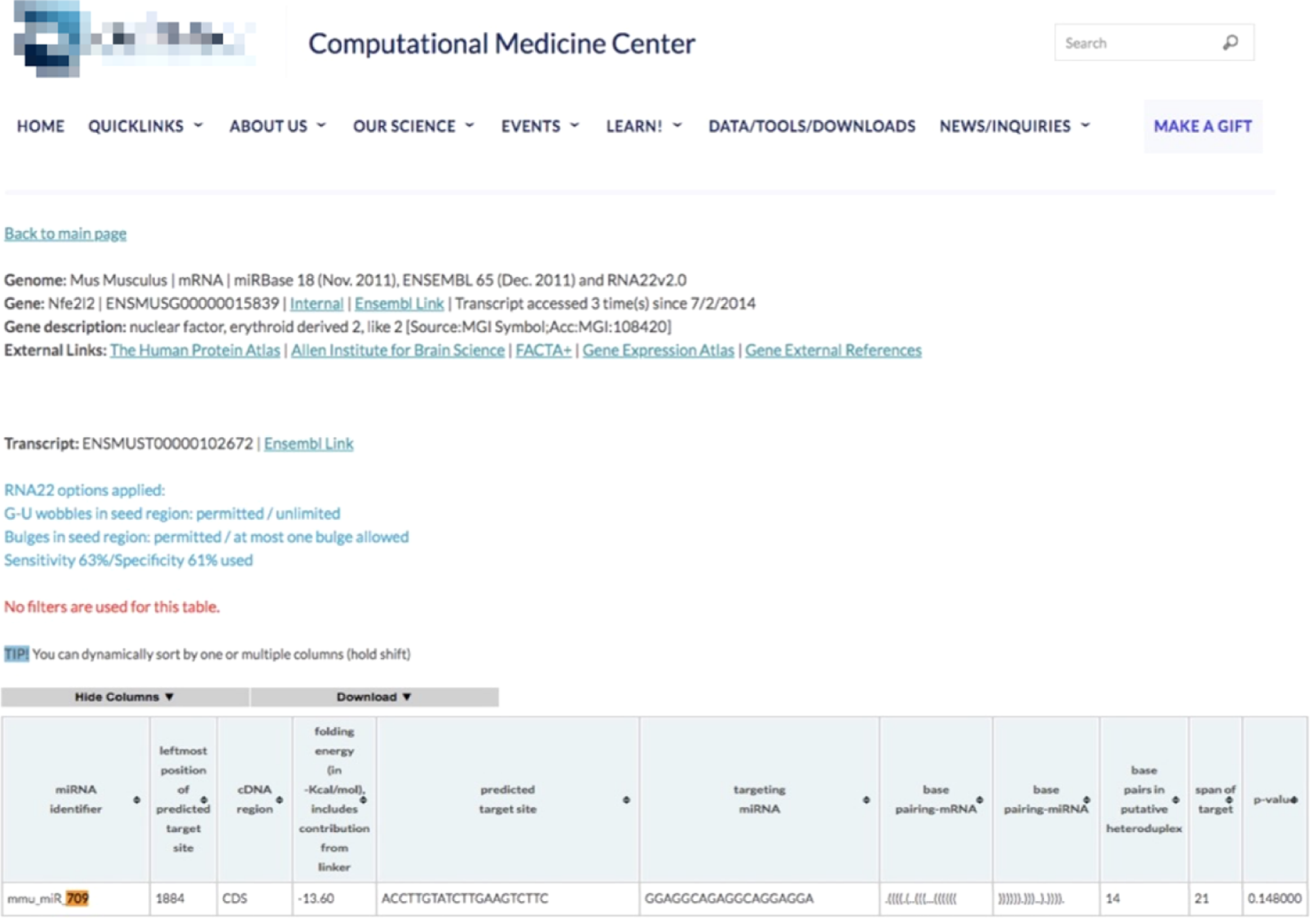
miR-709 interacted with NFE2L2 mRNA to promote its posttranscriptional degradation. Based on RNA22 prediction, the CDS of NFE2L2 contains one potential seed site for miR-709 targeting.

To sort miRNA potential and candidates miRNA target genes for DN, an online database search for miRNA target genes was conducted using tools such as miRanda, RNA22, and TargetScan. According to the analytical results of RNA22, the coding sequence (CDS) of human *NFE2L2* contains a potential seed site targeted by hsa-miR-709 targeting, indicating that *NFE2L2* is a potential target gene for miR-709 (Figure 8). Because miR-709 was identified as one of the top 10 upregulated genes identified in our miRNA microarray validation and because NRF2 has been described to play a role in cardiovascular disease, we investigated whether miR-709 mediates cellular oxidative stress by targeting *NFE2L2* in HG + PA (which mimic glucolipotoxicity state)-treated MES-13 cells.

### Utilizing an miR-709 Inhibitor Ameliorates Cellular Oxidative Stress

**Figure 9:**
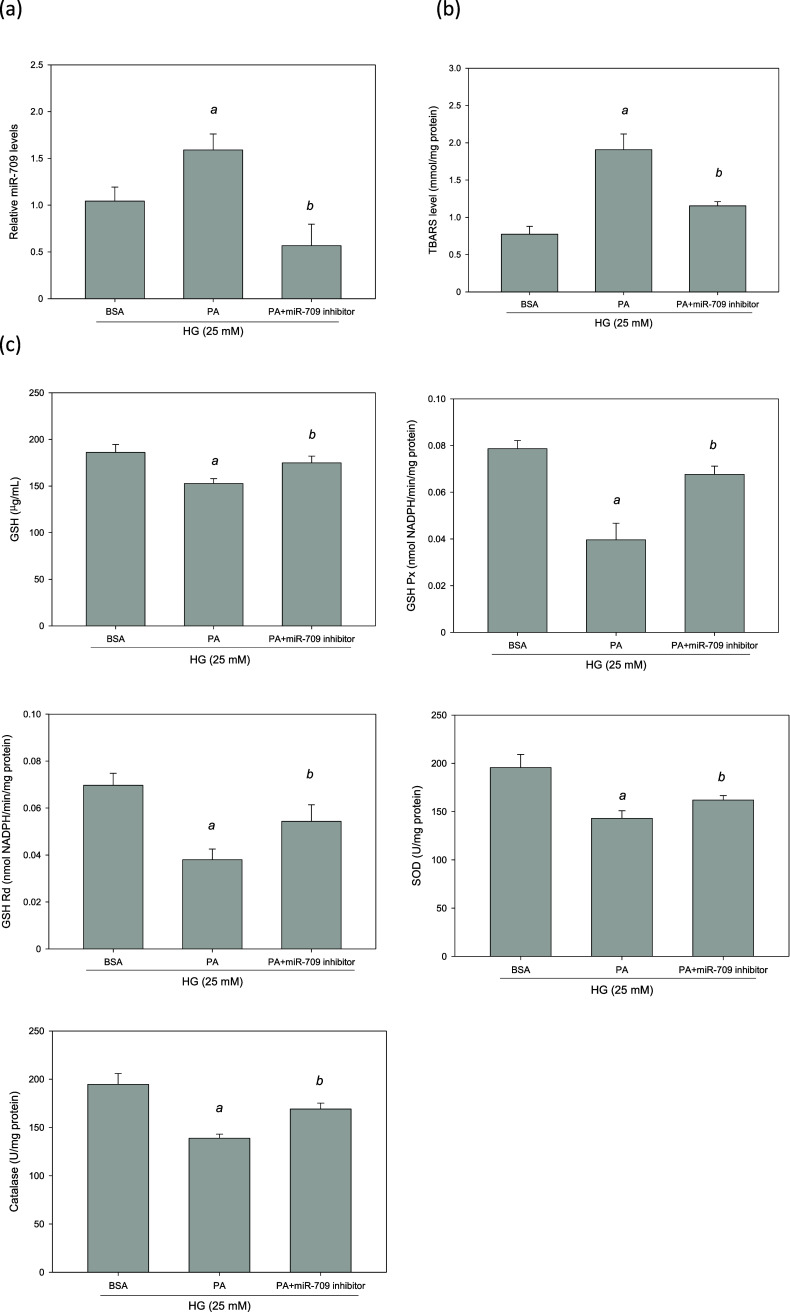
Suppression of miR-709 expression ameliorated PA-induced oxidative stress in HG-treated MES-13 cells. MES-13 cells were transfected with an miR-709 inhibitor or NC for 48 h in the presence of PA and HG. (a) Real-time PCR analysis of miR-709 expression. (b) Intracellular TBARS level. (c) The activities of antioxidant enzymes. ^a^, *p* < 0.05 compared with BSA control group. ^b^, *p* < 0.05 compared with HG + PA group.

MES-13 cells were used as a model and were treated with PA in the presence of HG. This significantly increased the levels of TBARSs (*p* < 0.05, Figure 9b). However, it significantly reduced the levels of renal antioxidant enzymes, including GSH Px, GSH, SOD, GSH Rd and catalase (*p* < 0.05, Figure 9c). Therefore, an HG + PA MES-13 cell model was recognized to mimic the status of excessive oxidative stress in concurrent T2DM and nephropathy. In this model, miR-709 was overexpressed by 54% in the presence of PA in HG-treated MES-13 cells (*p* < 0.05, Figure 9a). To identify the role of miR-709, MES-13 cells were transfected with a miR-709 inhibitor for 48 h. As presented in Figure 9a, this miR-709 inhibitor significantly suppressed the HG + PA-induced overexpression of miR-709. It also significantly reduced the levels of TBARSs (*p* < 0.05, Figure 9b). However, it also significantly increased the levels of antioxidant enzymes, including GSH Px, GSH, SOD, GSH Rd and catalase (*p* < 0.05, Figure 9c). These findings indicate that in the presence of HG and PA, miR-709 inhibition ameliorates lipid peroxidation, reduces cellular oxidative stress, and increases the activity of renal antioxidant enzymes in glomerular mesangial cells.

### Utilizing miR-709 Mimics Induced Cellular Oxidative Stress

**Figure 10:**
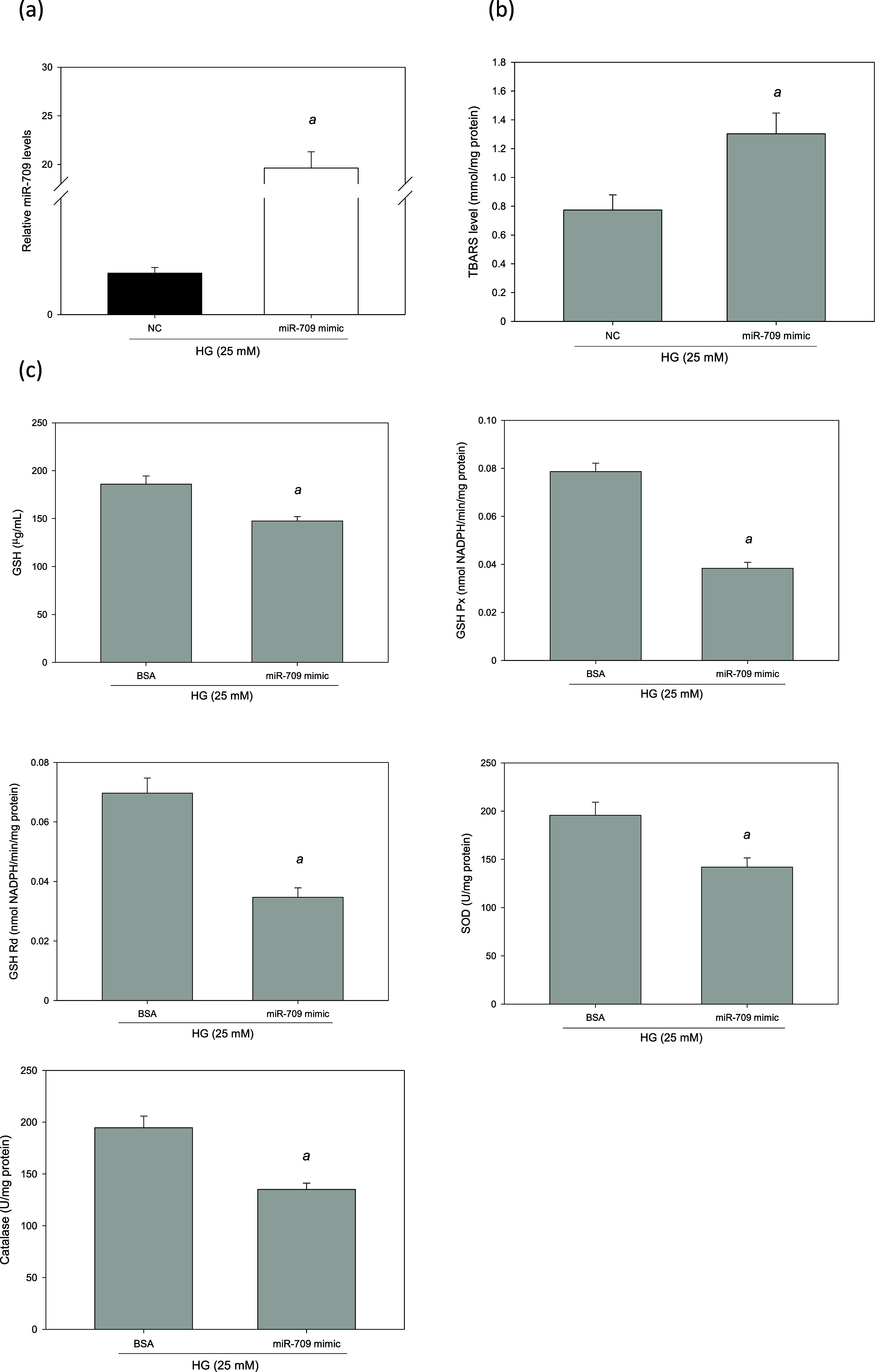
miR-709 induced cellular oxidative stress in HG-treated MES-13 cells. MES-13 cells were transfection with an miR-709 mimic or NC for 48 h. (a) Real-time PCR analysis of miR-709 expression. (b) Intracellular TBARS level. (c) The activities of antioxidant enzymes. ^a^, *p* < 0.05 compared with NC or BSA control group.

To determine whether miR-709 can induce a cellular oxidative response, HG-treated MES-13 cells were transfected with miR-709 mimics for 48 h. The finding indicated that miR-709 was overexpressed by 1745% (*p* < 0.05, Figure 10a). The miR-709 mimics significantly increased the levels of TBARSs by 69% (*p* < 0.05, Figure 10b). However, they significantly reduced the antioxidant enzymes levels, including GSH Px, GSH, SOD, GSH Rd and catalase (*p* < 0.05, Figure 10c). These results indicate that when the expression of miR-709 increases, the levels of lipid peroxidation and cellular oxidative stress in glomerular mesangial cells also increase. Overall, the effects of miR-709 mimics are similar to those observed in the presence of PA in HG-treated MES-13 cells, and they are consistent with the outcomes of our in vivo experiment conducted on HFD-fed db/db mice.

### Utilizing miR-709 Mediates Oxidative Responses by Straight Targeting *NFE2L2*

**Figure 11:**
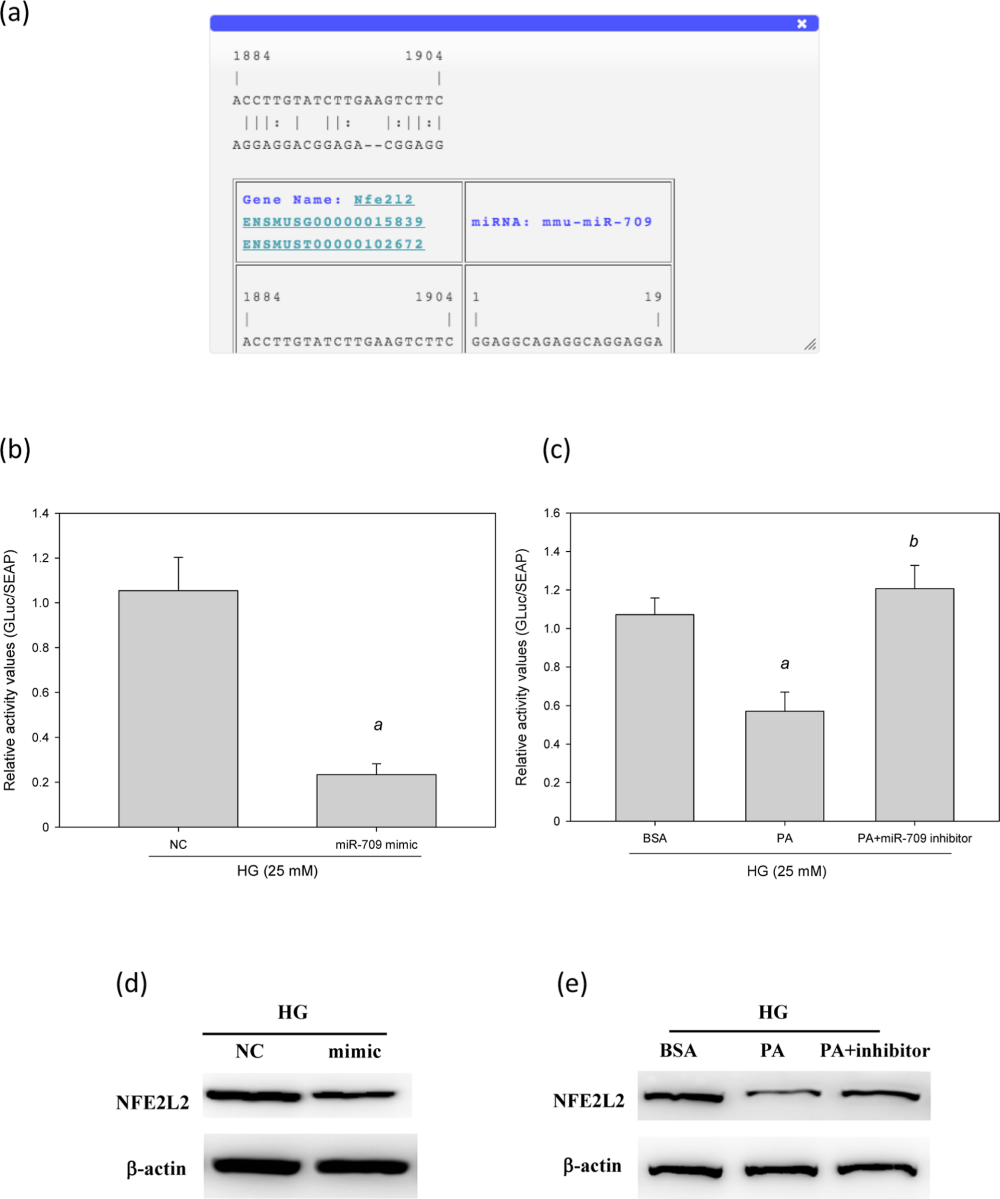
miR-709 mediated cellular oxidative stress by directly targeting NFE2L2 in HG-treated MES-13 cells. (a) A potential interacting site in the human NFE2L2 was predicted by RNA22. (b,c) Luciferase reporter plasmids (1 μg) containing the wild type of human NFE2L2 were cotransfected with 40 nM control mimic (NC), 40 nM miR-709, 20 pM control inhibitor (PA) and 20 pM miR-709 inhibitor into MES-13 cells plated in 12-well plates. After 24 h, the luciferase activity was measured using the Secrete-Pair GLuc Assay Kit. (d,e), Western blotting analysis of NFE2L2 in MES-13 cells transfected with an miR-709 mimic (d) or miR-709 inhibitor (e). ^a^, *p* < 0.05 compared with normal control group (NC or BSA group). ^b^, *p* < 0.05 compared with HG + PA group.

According to the analytical results of RNA22, the promoter of human *NFE2L2* holds one potential targeting seed site for hsa-miR-709 (Figure 11a). To determine whether *NFE2L2* is a true target of miR-709 and analyze the communication among *NFE2L2* mRNA and miR-709, we produced a *GLuc* reporter plasmid containing the 3′ untranslated region (3′-UTR) of human *NFE2L2* with a miR-709 binding site. A reporter assay performed in MES-13 cells revealed that the overexpression of miR-709 induced by the miR-709 mimics reduced the activity of luciferase by 78% (*p* < 0.05, Figure 11b). Likewise, cotransfection with a control inhibitor (PA) induced the expression of miR-709, thus reducing the activity of luciferase by 47%. After the expression of miR-709 was suppressed by an miR-709 inhibitor in the MES-13 cells, the activity of luciferase increased by 111% (*p* < 0.05, Figure 11c). According to the results of Western blotting, the miR-709 mimics downregulated the expression of *NFE2L2* (Figure 11d), whereas the miR-709 inhibitor had the opposite effect (Figure 11e). These findings indicate that hsa-miR-709 mediates cellular oxidative responses by straight targeting *NFE2L2* in MES-13 cells.

### GA Reduces HG + PA-Induced Oxidative Stress by Downregulating the Expression of miR-709

**Figure 12:**
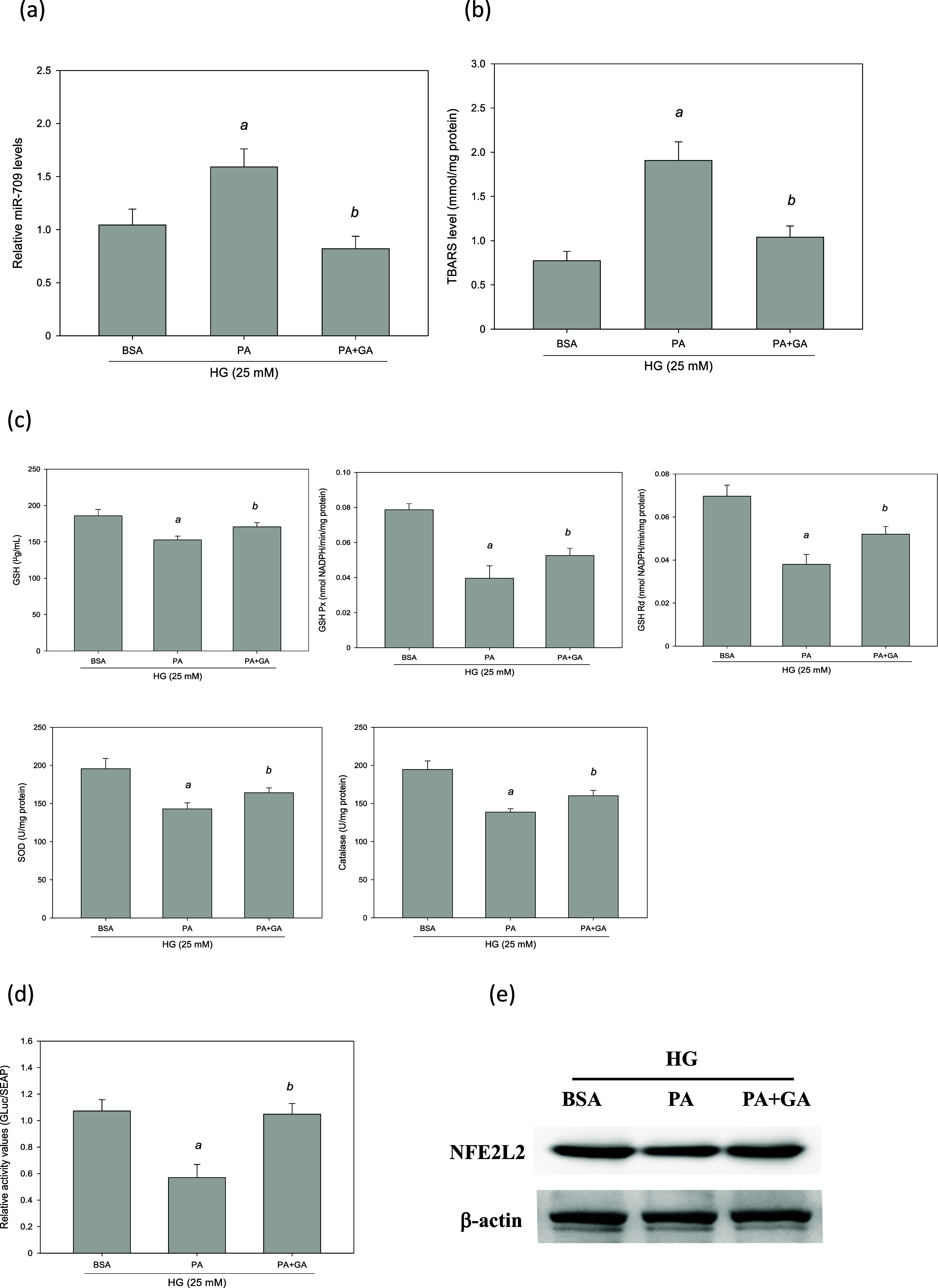
GA-induced reduction in renal oxidative stress is associated with suppression of miR-709 expression in MES-113 cell. MES-13 cells were treated with 10 μM GA for 24 h in the presence of PA. (a) Real-time PCR analysis of miR-709 expression. (b) Intracellular TBARS level. (c) The activities of antioxidant enzymes. (d) Luciferase activity. (e) Western blotting analysis of NFE2L2 in MES-13 cells treated with HG + PA and GA. ^a^, *p* < 0.05 compared with normal control group (NC or BSA group). ^b^, *p* < 0.05 compared with HG + PA group.

To determine whether GA can reduce HG + PA-induced oxidative stress in MES-13 cells, HG-treated MES-13 cells were treated with GA in the company with PA. After treatment with PA, the levels of TBARSs increased. However, after treatment with GA, the levels of TBARSs significantly decreased (*p* < 0.05, Figure 12b). Furthermore, after treatment with PA, the antioxidant enzymes level, including GSH Px, GSH, SOD, GSH Rd and catalase, significantly decreased. However, after treatment with GA, the levels of renal antioxidant enzymes significantly increased (*p* < 0.05, Figure 12c). These findings indicate that GA can reduce HG + PA-induced oxidative stress in MES-13 cells by ameliorating lipid peroxidation and enhancing oxidative responses.

To establish whether GA can reduce oxidative stress by moderating miR-709 in MES-13 cells, the expression levels of miR-709 were measured. The results showed that GA significantly downregulated the expression of miR-709, which was originally upregulated by HG and PA (*p* < 0.05, Figure 12a), whereas it upregulated the expression of *NFE2L2* (Figure 12e). *NFE2L2* luciferase reporter gene analysis revealed that GA meaningly increased the activity of luciferase, which was originally reduced by HG and PA, by 184% (*p* < 0.05, Figure 12d). These findings indicate that GA effectively reduces lipid peroxidation, enhances renal antioxidant enzyme activity, upregulates the expression of *NFE2L2*, and reduces HG + PA-induced oxidative stress by downregulating the expression of miR-709 and straight targeting *NFE2L2* in MES-13 cells.

## Discussion

We explored how GA affects glucolipotoxicity-induced nephropathy by downregulating the expression of miR-709 and targeting the expression of *NFE2L2* in HFD-fed db/db mice. We discovered that miR-709 shows a key role in the metabolism dysregulation and oxidative responses associated with DN in HFD-fed diabetic mice. We also recognized *NFE2L2* as a straight target of miR-709 and reported that GA ameliorates DN and downregulates the expression of miR-709 in murine glomerular mesangial (MES-13) cells. These findings suggest that miR-709-NFE2L2 is a new pathway involved in DN. Overall, these insights could facilitate the development of novel therapies for DN by targeting upstream molecules at the miRNA level.

DN is a chronic kidney disorder that can progress to end-stage renal disease due to diabetes-related complications. Its key features include the buildup of extracellular matrix (ECM) proteins, thickening of the cell expansion, glomerular basement membrane, mesangial and kidney hypertrophy. In patients with DN, the concentration of TGF-β, a key regulator of ECM genes, increases in mesangial cells.^18^

In this study, we used HFD-fed db/db mice as a diabetic mouse model, and we discovered that an HFD substantially affected renal tissue and resulted in basement membrane thickening in the glomerulus and a marked deposition of collagen, as determined. The HFD-fed mice shown decreased renal function; increased plasma levels of BUN, creatinine, cholesterol, and triglycerides; increased levels of insulin; and increased body weight. Histopathological examination also exposed an amplified accumulation of polymorphonuclear leukocytes and adipocytes, leading to DN; this finding is reliable with those of previous studies.^11,35^ Overall, these findings indicate that the HFD resulted in impaired renal function and hyperinsulinemia and induced diabetic kidney disease accompanied by a decline in renal function, which is consistent with the findings of a previous study.^35^ On the other hand, previous studies on GA have primarily focused on glucose after meals (Glucose AC), observing that fasting blood glucose levels decrease due to the inhibition of glycogenolysis.^36^ We utilized the db/db mouse model to simulate postprandial glucose (Glucose PC). These mice are derived from inbreeding heterozygous C57BL/KsJ mice and are characterized by a deficiency in functional leptin receptors. Leptin is essential for regulating blood glucose levels by suppressing glucagon and corticosterone production, enhancing glucose uptake, and reducing hepatic glucose production. In our db/db mouse model, the absence of functional leptin receptors eliminates these glucoregulatory effects. As a result, we maintained a glucotoxic environment throughout the entire experimental period to mimic Glucose PC, which, as expected, did not main to a reduction in blood glucose levels.

According to the literature, any increase in serum or plasma glucose levels can result in excessive production of reactive oxygen species, which play a main role in the pathogenesis of diabetic complications.^14^ The TBARS assay can be used to regulate the extent of lipid peroxidation. In this study, we discovered that the levels of TBARSs in the kidneys were meaningfully higher in the HFD group than in the db group. However, treatment with GA reduced the levels of TBARSs.

A wide range of natural products has been utilized in the treatment of metabolic disorders, with numerous bioactive compounds, including plant phenolics, playing a significant role, have been reported to have positive effects on health. GA is a type of phenolic acid commonly found in various fruits and traditional herbal remedies. It has antioxidative, anticancer, anti-inflammatory, and antimicrobial effects.^48^ In this study, treatment with GA significantly improved renal parameters and increased the levels of uric acid. In HFD-fed diabetic mice, treatment with GA for 12 weeks ameliorated kidney damage by reducing basement membrane thickening and collagen deposition. Taken together, these findings indicate that GA has a renoprotective effect, ameliorates nephropathy by enhancing renal function, and protects against hyperinsulinemia and glomerular fibrosis, consistent with the findings of previous studies.^1,11^

GA is a common antioxidant in tea formulations. Its antihyperglycemic, anti-lipid peroxidation, and antioxidant effects may be responsible for its renoprotective effect. In our DN model, GA reduced oxidative stress and increased the levels of GSH, GSH Rd, GSH Px, and glutathione S-transferase (GST) in the renal tissue of HFD-fed db/db mice. Moreover, treatment with GA significantly improved hyperlipidemia, decreased lipid peroxidation and abnormal lipid accumulation, enhanced antioxidant enzyme activity, and suppressed the activity of lipogenic enzymes in db/db mice fed a HFD. These findings show that GA can ameliorate HFD-induced DN by influencing lipid peroxidation and oxidative stress and regulating fatty acid synthesis and lipid balance. Further study is required to explore the molecular mechanisms underlying the therapeutic effects of GA on diabetes-related complications, with an emphasis on oxidative damage in renal tissue.

According to a previous study, GA can reduce inflammation and oxidative stress in diabetic rats by modulating the expression of miRNAs.^19,32^ In this study, we studied whether GA exerts a protective effect by modifying miRNAs involved in renal lipid metabolism. In in vitro experiment, we discovered that the GA-induced decrease in intracellular triglycerides and lipogenic enzymes was mediated by suppression of miR-709 expression in renal tissue. These findings indicate that GA can function as a therapeutic agent against DN by downregulating the expression of miR-709.

miRNAs regulate gene expression and are often dysregulated in metabolic disorders such as DN. Studying cell-specific miRNA expression or circulating miRNA profiles can offer valuable insights and aid in the development of diagnostic tools for diseases.^4,27^ Analyzing miRNA signatures in cells or blood samples can also aid in differentiating patients with DN, which can enable more targeted and accurate diagnosis. In addition, miRNAs hold potential as therapeutic targets for treating metabolic disorders.

Multiple studies have indicated that miRNAs play a key role in the development of DN. For example, in diabetic glomeruli, the expression of miRNA-192 significantly increases, and miRNA-192 regulates the induction of collagen type 1 α2 through TGF-β.^18^ According to the results of in vitro and in vivo experiments, inhibiting the expression of miR-29c mitigates the apoptosis of kidney podocyte cells and reduces the accumulation of ECM proteins in renal cells.^24^ In high-sugar environments, the expression of miR-377 increases in human and mouse kidney mesangial cells, which indirectly stimulates the expression of fibronectin, a kidney fibrosis indicator protein.^45^ In glomerular mesangial cells cultured in a high-sugar environment and in the glomeruli of streptozotocin-induced diabetic rats, the expression of miR-27 significantly increases. When the expression of miR-27 decreases, the proliferative effect of mesangial cells in the glomeruli also decreases, reducing the accumulation of ECM proteins and ameliorating proteinuria in rats.^46^

Bioinformatics is an approach in which computational tools are used to analyze biological data. In addition, microarray analysis is a technique used to identify specific miRNAs associated with pathways such as carcinogenesis that may serve as indicators or signatures of disease.^33,40^ In this study, we employed miRNA microarray validation to examine 1415 miRNAs, revealing significant changes in the expression of several miRNAs in db/db mice treated with HFD. Therefore, we analyzed the top 10 upregulated and downregulated miRNAs that responded to the HFD. This enabled identification of putative miRNA binding sites in sequences of interest and aided in determining the identity of target miRNAs.^29^ Specific binding was observed between miR-709a and the luciferase construct, confirming posttranscriptional modification of miR-709a-5p in *NFE2L2*.

miRNAs play an essential role in the regulation of gene expression. Previous studies examining the role of miRNAs in gene expression regulation have primarily focused on their target sites located in the 3′-UTR of mRNAs. However, multiple studies have indicated that miRNAs can also degrade mRNAs and affect protein transcription and translation by binding to the CDS and 5′-UTR of mRNAs.^13,15^ Analysis of the luciferase reporter gene in *NFE2L2* indicates that in human or renal glomerular cells, miR-709 can directly affect the activity of *NFE2L2* luciferase, confirming that miRNAs regulate gene expression by binding to other positions in the 3′-UTR.

According to the literature, miR-709 originates from chromosome 8 and is expressed in various mouse tissues, including the brain, thymus, heart, lungs, liver, spleen, kidneys, adipose tissue, and testes.^38,39^ In adipocytes, miR-709 affects the differentiation of adipocytes by inhibiting the expression of GSK3β.^6^ In addition, miR-709 regulates the inflammatory response induced by lipopolysaccharides by targeting GSK3β and upregulating the expression of β-catenin.^21^

In AKI kidney cells, the expression of miR-709 increases with the severity of injury. Overexpression of miR-709 results in mitochondrial dysfunction and cell death, whereas its inhibition is associated with improved outcomes. In addition, miR-709 targets TFAM (mitochondrial transcriptional factorA), a key factor in mitochondrial function. Inhibiting miR-709 reduces kidney injury and dysfunction in mice. These findings suggest that miR-709 is a potential target for AKI treatment.^12^

In mice with DN, high miR-709 expression is associated with kidney damage. Inhibiting miR-709 can reduce oxidative stress in kidney cells and increase the activity of antioxidant enzymes, thereby protecting the kidneys. GA is a polyphenol that can ameliorate DN by inhibiting the expression of miR-709, which in turn increases oxidative stress and reduces antioxidant enzyme activity, leading to kidney damage.

Oxidative stress is the main cause of complications in diabetes, including DN. Current antioxidant treatments for DN involve a range of phytochemicals, including dietary antioxidants, resveratrol, curcumin, and alpha-lipoic acid preparations.^7^*NFE2L2* is a key regulator of redox balance and detoxification in the body. Natural compounds extracted from plants and vegetables can activate *NFE2L2*, thus increasing the activity of antioxidant enzymes and mitigating damage resulting from oxidative stress and high blood sugar.^41^*NFE2L2* is a transcription factor that promotes antioxidant activity by controlling the expression of genes responsible for producing antioxidant enzymes, including glutathione peroxidase (GSH Px), GST, superoxide dismutase (SOD), heme oxygenase-1, and NAD(P)H oxidoreductase-1.^20^*NFE2L2* protects the kidneys against diabetic kidney disease by suppressing oxidative stress and inflammation.^23^

In patients with DN, miR-709 inhibits the expression of the antioxidant transcription factor *NFE2L2*, which increases oxidative stress in renal cells and reduces the activity of intracellular antioxidant enzymes. Previous studies have shown that miRNAs can be utilized noninvasively to monitor the diagnosis and progression of kidney diseases. For example, the injection of a miR-709 antagomir was found to attenuate cisplatin-induced AKI in mice by mitigating mitochondrial dysfunction through the regulation of its target gene, mitochondrial transcription factor A (TFAM).^12^ Furthermore, NFE2L2 plays a critical role in defending against oxidative stress by regulating and activating the intracellular antioxidant system to neutralize ROS and maintain redox homeostasis.^47^ Studies have demonstrated that NFE2L2 provides protective effects in diabetes, both in vitro and in vivo, by inhibiting TGF-β1 and reducing extracellular matrix production.^49^ Taken together, our findings indicate that overexpression of miR-709 in the kidneys results in excessive lipid accumulation in the kidney cells, indicating that the presence of excessive amounts of miR-709a-5p may be detrimental to the organism. Therefore, miR-709a-5p modulates the development and progression of DN, and GA significantly ameliorates DN by downregulating the expression of miR-709a-5p and targeting *NFE2L2* in diabetic mice. In this study, we confirmed that in db/db mice, GA enhances the regulation of miRNAs, reduces glucolipotoxic effects in the kidneys, and slows the progression of complications associated with DN. This improvement in glucose and lipid toxicity management through miRNA modulation contributes to the therapeutic potential of GA in DN.

## Conclusion

In this study, we discovered that miR-709a-5p shows a key role in the regulation of metabolism in diabetic kidneys and is upregulated in the renal tissue of mice with DN. Overexpression of miR-709a-5p increases oxidative stress and the levels of lipogenic enzymes, which in turn contribute to DN by modulating the suppression of its target *NFE2L2*. In diabetic kidney disease, miR-709a-5p inhibitors may have a therapeutic effect. GA and *NFE2L2*-activating agents can downregulate renal miR-709a-5p expression and thus may be useful in the treatment of DN. In HFD-fed db/db mice, GA exerts positive effects on glucolipotoxicity-induced DN by regulating the levels of miRNAs. These effects are evidenced by enhancements in body weight, insulin sensitivity, blood lipid profiles, and renal function. Overall, GA holds possible as a therapeutic agent for the running of DN; this potential should be further explored in human clinical trials.
